# Semi-Global Matching Assisted Absolute Phase Unwrapping

**DOI:** 10.3390/s23010411

**Published:** 2022-12-30

**Authors:** Yi-Hong Liao, Song Zhang

**Affiliations:** School of Mechanical Engineering, Purdue University, West Lafayette, IN 47907, USA

**Keywords:** phase-shifting, phase unwrapping, high speed, Semi-Global Matching, digital image correlation, 3D reconstruction

## Abstract

Measuring speed is a critical factor to reduce motion artifacts for dynamic scene capture. Phase-shifting methods have the advantage of providing high-accuracy and dense 3D point clouds, but the phase unwrapping process affects the measurement speed. This paper presents an absolute phase unwrapping method capable of using only three speckle-embedded phase-shifted patterns for high-speed three-dimensional (3D) shape measurement on a single-camera, single-projector structured light system. The proposed method obtains the wrapped phase of the object from the speckle-embedded three-step phase-shifted patterns. Next, it utilizes the Semi-Global Matching (SGM) algorithm to establish the coarse correspondence between the image of the object with the embedded speckle pattern and the pre-obtained image of a flat surface with the same embedded speckle pattern. Then, a computational framework uses the coarse correspondence information to determine the fringe order pixel by pixel. The experimental results demonstrated that the proposed method can achieve high-speed and high-quality 3D measurements of complex scenes.

## 1. Introduction

Three-dimensional (3D) shape measurement has applications in many fields, such as forensic science, medical surgery, and robotics. In the fields such as robotics, the measuring scene is usually dynamic. Therefore, in addition to measurement accuracy, measurement speed is also a critical factor for alleviating motion artifacts. The widely adopted 3D measurement techniques include stereo vision, time-of-flight, and phase-based methods. Although stereo vision and time-of-flight methods usually have higher capturing speeds, phase-based methods have the advantages of robustness and accuracy. They can provide dense 3D point clouds with high spatial resolutions. Over the years, numerous phase-based methods have been introduced including the Fourier method [1], the Windowed Fourier method [2], and the phase-shifting methods [3]. These methods are capable of retrieving phase information. However, the retrieved phases are wrapped between −π and π with 2π discontinuities. Therefore, phase unwrapping algorithms have to be employed to eliminate these discontinuities.

Phase unwrapping algorithms essentially find the integer multiple of 2πs to add or subtract. These integers are usually called the *fringe order*, *K*. There are roughly two types of phase unwrapping algorithms—spatial phase unwrapping algorithms and temporal phase unwrapping algorithms. Spatial phase unwrapping algorithms detect the 2π discontinuities and determine the fringe order *K* of each pixel by analyzing the phase values of the neighboring pixels on the wrapped phase map itself. Some of the approaches are scan-line unwrapping methods, quality-guided methods [4,5,6], and multi-anchor unwrapping methods [7]. Regardless of the speed of spatial phase unwrapping methods, they usually generate relative phase maps because the phase is unwrapped with respect to a starting point on the wrapped phase map. Therefore, the phase and the 3D points reconstructed are relative instead of absolute. On the other hand, temporal phase unwrapping algorithms determine the fringe order by referring to information from additional captured images. Since each pixel is unwrapped independently, the temporal phase unwrapping algorithm can generate absolute phase maps. Over the years, numerous temporal phase unwrapping methods have been developed including the binary-coding method [8], the gray-coding method [9], the multi-wavelength method [10,11,12], and the phase encoding method [13]. All these methods can retrieve absolute phase maps, yet they require the capturing of additional images which will slow down the measuring speed and is not desirable for high-speed applications.

To address the measuring speed reduction issue, An et al. [14] developed a phase unwrapping algorithm that utilizes the geometric constraint between the camera and the projector on a structured light system. An artificial absolute phase map is generated to assist in absolute phase unwrapping. This method does not require additional images. However, in order to correctly acquire the absolute phase, the minimum depth of the measuring object has to be known. Additionally, there is a measuring range limitation which depends on the spatial span of the fringe period and the angle between the projector and the camera. An and Zhang [15] combined the binary statistical matching with the phase-shifting method. They match the binarized statistical patterns captured by the camera with the ideal computer-generated projector image to generate the disparity map. The disparity map is further refined and used to obtain the final unwrapped phase. This method only requires the projection of one additional image and does not have the limitations of the previous method. However, the projector and camera usually have different sensor sizes and lenses, and the computer-generated images do not undergo the lens effects that the camera-captured images do. Hence, the projector or camera images have to be cropped and down-sampled (or up-sampled) before matching to match the field-of-view and the resolution, and the matching result requires extensive hole filling and refinement to correct the phase value. Zhang et al. [16] captured speckle-embedded fringe patterns of reference planes at different distances to generate a wrapped phase-to-height lookup table (LUT). Then the absolute spatial height of the measured object can be reconstructed by looking up the LUT with the speckle correlation. This method needs only four speckle-embedded fringe patterns and the wrapped phase constraint improves the LUT computational efficiency. However, this method requires the capturing of reference planes at many different heights and the measuring range is limited by the LUT.

Researchers have also attempted to add extra cameras to the standard single-camera and single-projector structured light system. Stereo-assisted phase-shifting profilometry [17,18,19,20,21,22,23] has been proposed in past few years. With extra cameras, conventional stereo vision techniques can be used along with the phase information to assist image correspondence establishment. However, adding extra cameras increases the costs and the complexity of hardware and algorithm development [24]. In addition, only the region that can be simultaneously observed by all cameras and projectors can be measured, which further limits the field of view. Therefore, in our previous work [25], we incorporated the digital image correlation (DIC) with the phase-shifting method on a single-camera and single-projector structured light system. Three phase-shifted fringe images and one speckle pattern are projected. The inverse-compositional Gauss–Newton algorithm (IC-GN) [26] is used to establish the correspondence between the pre-captured speckle image of a white surface and the captured speckle image of the measuring object. Then we developed a computational framework to use the correspondence to assist high-accuracy absolute phase retrieval. This method has been proven successful. However, although we have significantly improved the IC-GN algorithm efficiency by using the wrapped phase and epipolar constraint, the algorithm is still time-consuming. Given this, we only perform IC-GN on interval pixels and use the resultant deformation vector to complete the remaining correspondence.

In this research, we propose a phase unwrapping method on a single-camera and single-projector structured light system that combines Semi-Global Matching (SGM) [27] with the phase-shifting method to perform high-accuracy 3D measurements. Similar to our previous method, the wrapped phase map is obtained from the phase-shifted patterns. Instead of the IC-GN algorithm, the SGM algorithm is used to establish the coarse correspondence between the camera coordinate and the projector coordinate. Then the coarse correspondence is used to unwrap the wrapped phase. Finally, the spatial phase unwrapping is applied locally to each pixel with the SGM correspondence error to generate the final absolute phase map. The experiments verified the success of our proposed method. The computational speed is significantly increased compared to the IC-GN algorithm. Therefore, instead of interval pixel search, we can conduct SGM on every pixel which increases the number of pixels that have correspondence results. In addition, because the SGM algorithm improves the result of the correspondence search in our experiments, the proposed method has the ability to further reduce the number of patterns projected to three by allowing us to embed the speckle pattern into the three-step phase-shifted patterns, which is desirable for high-speed absolute 3D shape measurement.

## 2. Materials and Methods

### 2.1. Speckle-Embedded Three-Step Phase-Shifting Algorithm

The three-step phase-shifted fringe images with equal phase shifts can be mathematically written as

(1) $$I_{k}\left(x,y\right)=I^{\prime }\left(x,y\right)+I^{\prime \prime }\left(x,y\right)\cos\left[\phi \left(x,y\right)-\frac{2\pi k}{3}\right],\;k=1,2,3,$$

where (x,y) is the pixel coordinate, $I^{\prime }\left(x,y\right)$ is the average intensity, $I^{\prime \prime }\left(x,y\right)$ is the intensity modulation and ϕ(x,y) is the phase to be solved. Speckle patterns can be embedded into the phase-shifted fringe patterns using Lohry and Zhang’s method [21]. The speckle-embedded fringe images can be written as

(2) $$\hat{I_{k}}\left(x,y\right)=I^{\prime }\left(x,y\right)+I_{p}\left(x,y\right)I^{\prime \prime }\left(x,y\right)\cos\left[\phi \left(x,y\right)-\frac{2\pi k}{3}\right],\;k=1,2,3,$$

where $I_{p}\left(x,y\right)$ is randomly generated intensity ranging from 0.5 to 1. The embedded speckle pattern can be recovered by

(3) $$\hat{\gamma }=\frac{I_{p}\left(x,y\right)I^{\prime \prime }\left(x,y\right)}{I^{\prime }\left(x,y\right)}=\frac{\hat{I^{\prime \prime }}\left(x,y\right)}{I^{\prime }\left(x,y\right)}=\frac{\sqrt{3{\left(\hat{I_{1}}-\hat{I_{3}}\right)}^{2}+{\left(2\hat{I_{2}}-\hat{I_{1}}-\hat{I3}\right)}^{2}}}{\hat{I_{1}}+\hat{I_{2}}+\hat{I_{3}}},$$

and the phase ϕ(x,y) can be solved by

(4) $$\phi \left(x,y\right)=\tan^{-1}\left[\frac{\sqrt{3}\left(\hat{I_{1}}-\hat{I_{3}}\right)}{2\hat{I_{2}}-\hat{I_{1}}-\hat{I_{3}}}\right].$$

The speckle-embedded fringe images are 8-bit grayscale images. We convert the 8-bit grayscale patterns to 1-bit binary patterns by applying MATLAB’s dithering function (Floyd–Steinberg’s error diffusion dither algorithm [28]). Although dithering lowers the fringe pattern quality, it can significantly increase the pattern projection rate on a digital light processing (DLP) projector, thus the 3D measurement speed.

The phase map solved from Equation (4) ranges from −π to π and has 2π discontinuities between each phase period because of the arctangent operation, which we call the wrapped phase. Conventionally, to eliminate the 2π discontinuities, a temporal or spatial phase unwrapping algorithm is applied. The mathematical relationship between a wrapped phase and an unwrapped phase is

(5) Φ(x,y)=ϕ(x,y)+2π×K,

where *K* is called the *fringe order*. The phase unwrapping algorithm is essentially finding the fringe order *K* of each pixel such that the 2π discontinuities are removed. In this research, we developed a phase unwrapping algorithm based on the pipeline in our previous work [25]. The proposed algorithm finds the low-accuracy absolute phase of each pixel in the scene automatically and uses it to unwrap the wrapped phase to obtain the high-accuracy absolute phase. Then, the high-accuracy absolute 3D geometry can be reconstructed. Instead of using the IC-GN algorithm, we employed the SGM algorithm in this work. The SGM algorithm improves the matching result in our experiments and significantly speeds up the computational process which allowed us to conduct a pixel-wise instead of grid points correspondence search.

### 2.2. Low-Accuracy Absolute Phase Extraction Using SGM

Prior to any 3D measurement, we projected and captured speckle-embedded three-step phase-shifted fringe patterns on a white flat surface. Then, we recovered the speckle pattern from the fringe images reflected by the white flat surface using Equation (3). We denote the $\hat{\gamma }$ retrieved from images of the white flat surface as

(6) $${\hat{I}}_{r}=\hat{\gamma }.$$

The hat (^) symbol means that the image is retrieved from the speckle-embedded three-step phase-shifted fringe patterns. We also projected two sets of fifteen phase-shifted fringe patterns onto the same white flat surface to obtain the horizontal and vertical phase maps, $\Phi_{r}^{h}$ and $\Phi_{r}^{v}$, using the multi-wavelength phase-shifting algorithm. There exists a unique mapping between ${\hat{I}}_{r}$, $\Phi_{r}^{h}$ and $\Phi_{r}^{v}$ because they are obtained from the same object with the same camera and projector positions. We save them for future use. It is important to note that we do not need to recapture the aforementioned images for any 3D measurement.

For a 3D measurement, we projected the same speckle-embedded three-step phase-shifted fringe patterns onto the object. Again, we recovered the speckle pattern from the fringe images reflected by the object using Equation (3). We denote the $\hat{\gamma }$ retrieved from images of the object as

(7) $${\hat{I}}_{o}=\hat{\gamma }.$$

**Figure 1:**
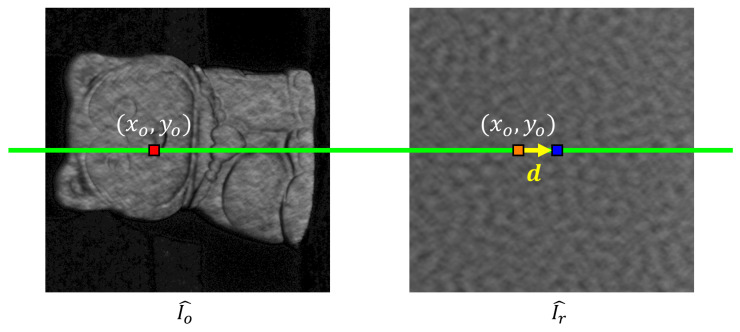
The definition of the disparity in this research. The disparity d is the pixel coordinate difference between the point of interest (POI) on ${\hat{I}}_{o}$ and its corresponding point on ${\hat{I}}_{r}$ found using SGM. (Red point: POI, blue point: corresponding point, green line: pre-computed epipolar line through absolute phase constraints, orange point: point on ${\hat{I}}_{r}$ with the same pixel coordinate as POI, yellow arrow: disparity vector d).

The wrapped phase ϕ of the object was calculated using Equation (4). Next, we used SGM [27] to obtain the correspondences between ${\hat{I}}_{r}$ and ${\hat{I}}_{o}$. SGM obtains the disparity map by finding the disparity that leads to the lowest total cost aggregated from multiple directions. Conventional stereo SGM searches between rectified stereo images within a predefined disparity range on the epipolar line. However, in our case, there was only one image from one camera at a time. Therefore, we have to find the epipolar points of ${\hat{I}}_{o}$ on ${\hat{I}}_{r}$ using the phase information. From the camera and the projector calibration data, we can find epipolar lines in the projector coordinate for pixels on ${\hat{I}}_{o}$. When we projected the multi-wavelength phase-shifted patterns, each pixel in the projector coordinate had been assigned a unique horizontal and vertical absolute phase value. Therefore, we can locate the points in ${\hat{I}}_{r}$ that are on the epipolar line by finding the pixel on each column (direction with small phase variation) of ${\hat{I}}_{r}$ that has coordinate ($\Phi_{r}^{h}$, $\Phi_{r}^{v}$) closest to the epipolar line. The pixel coordinate difference between the point of interest (POI) on ${\hat{I}}_{o}$ and its corresponding point on ${\hat{I}}_{r}$ found using SGM is the disparity vector d in this research. Figure 1 shows the definition of the disparity for the SGM algorithm in this research.

The cost $L_{r}\left(p,d\right)$ of pixel p aggregate along eight directions r at disparity vector d can be recursively defined as

(8) $$\begin{array}{cc} L_{r}\left(p,d\right)= & C\left(p,d\right)+\min(L_{r}\left(p-r,d\right),\\ \; & L_{r}\left(p-r,d-n\right)+P_{1},\\ \; & L_{r}\left(p-r,d+n\right)+P_{1},\\ \; & \min_{i}L_{r}\left(p-r,i\right)+P_{2})-\min_{k}L_{r}\left(p-r,k\right), \end{array}$$

where $P_{1}$ and $P_{2}$ are two constant parameters, with $P_{1}<P_{2}$. n is the disparity vector change tolerance for allowing smooth surface geometry changes. The cost function *C* we used is the Zero-mean Normalized Sum of Squared Differences (ZNSSD) criterion [29], which is insensitive to the potential scale and offset changes of the subset intensity. The ZNSSD coefficient can be expressed as

(9) $$C_{ZNSSD}=\sum_{\xi }{\left\{\frac{f\left(p+\xi \right)-\bar{f}}{\Delta f}-\frac{g\left(p+d+\xi \right)-\bar{g}}{\Delta g}\right\}}^{2},$$

where f(x) and g(x) are the grayscale values at $x={\left(x,y,1\right)}^{T}$ of ${\hat{I}}_{r}$ and ${\hat{I}}_{o}$. We only considered the valid pixels filtered by $\hat{\gamma }$ and $\hat{I^{\prime \prime }}$. $\xi ={\left(\Delta x,\Delta y,1\right)}^{T}$ is the local coordinates of the valid pixels in each subset and *N* is the total number of valid pixels in each subset. $\bar{f}=\frac{1}{N}\sum_{\xi }f(p+\xi )$ and $\bar{g}=\frac{1}{N}\sum_{\xi }g(p+d+\xi )$. $\Delta f=\sqrt{\sum_{\xi }{\left[f\left(p+\xi \right)-\bar{f}\right]}^{2}}$ and $\Delta g=\sqrt{\sum_{\xi }{\left[g\left(p+d+\xi \right)-\bar{g}\right]}^{2}}$.

We also incorporated SGM with the wrapped phase constraint we had. Theoretically, the corresponding points in ${\hat{I}}_{r}$ and ${\hat{I}}_{o}$ should have the same wrapped phase. The relation between the unwrapped phase map $\Phi_{r}^{h}$ and wrapped phase $\phi_{r}^{h}$ of ${\hat{I}}_{r}$ is

(10) $$\phi_{r}^{h}=\Phi_{r}^{h}\;\mathrm{mod}\;\left(2\pi \right).$$

**Figure 2:**
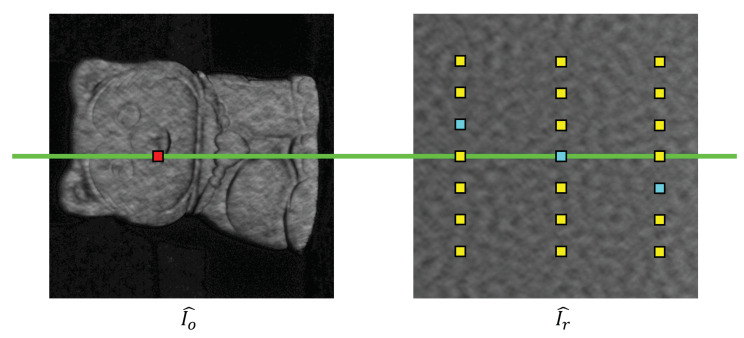
The schematic process of generating candidate corresponding points for the SGM algorithm. (Red point: POI, yellow points: offset pixels from epipolar points with wrapped phase constraint, light blue points: offset pixels with the smallest $C_{ZNSSD}$ on their column, green line: pre-computed epipolar line through absolute phase constraints).

By comparing $\phi_{r}^{h}$ and ϕ, we can further narrow down the epipolar points to those that have the same wrapped phase value. To reduce the impact of the system calibration error and phase noise, we calculated the $C_{ZNSSD}$ seven times, 0, ±3,±6,±9 offset pixels on the same column for all epipolar points after applying wrapped phase constraint, and chose the pixel with the smallest $C_{ZNSSD}$ to apply SGM search to determine coarse correspondence. Figure 2 shows the schematic process of generating candidate corresponding points for the SGM algorithm. Once the correspondences between ${\hat{I}}_{o}$ and ${\hat{I}}_{r}$ are established, we can extract the low-accuracy absolute phase map $\tilde{\Phi }$ of ${\hat{I}}_{o}$ using the mapping between ${\hat{I}}_{r}$ and $\Phi_{r}^{h}$.

### 2.3. High-Accuracy Absolute Phase Unwrapping

**Figure 3:**
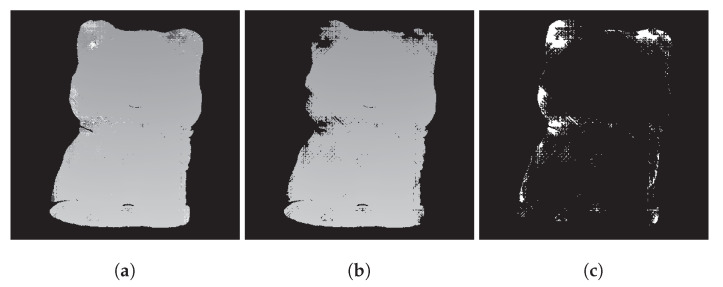
Example of determining the pixels to perform local spatial phase unwrapping on. (**a**) $\Phi_{1}$. (**b**) $\Phi_{2}$. (**c**) Pixels (white) that will be unwrapped by local spatial phase unwrapping.

The high-accuracy absolute phase Φ will be obtained by unwrapping the wrapped phase ϕ in the following steps. First, we determine the fringe order $\tilde{K}$ using the following equation,

(11) $$\tilde{K}\left(x,y\right)=round\left[\frac{\tilde{\Phi }-\phi }{2\pi }\right],$$

where round() is the rounding operator that obtains the closest integer to the value. We used $\tilde{K}\left(x,y\right)$ to generate unwrapped phase $\Phi_{1}$. Second, we removed the errors in $\Phi_{1}$ due to false SGM results at low signal-to-noise ratio areas and the abrupt surfaces by masking out the connected regions that had an area size lower than a threshold and obtained $\Phi_{2}$. Finally, we performed local spatial phase unwrapping [7] with respect to $\Phi_{2}$ on the pixels we masked out in the previous step to obtain the final high-accuracy absolute phase, Φ. Figure 3 shows examples of $\Phi_{1}$, $\Phi_{2}$, and the pixels we performed local spatial phase unwrapping on.

**Figure 4:**
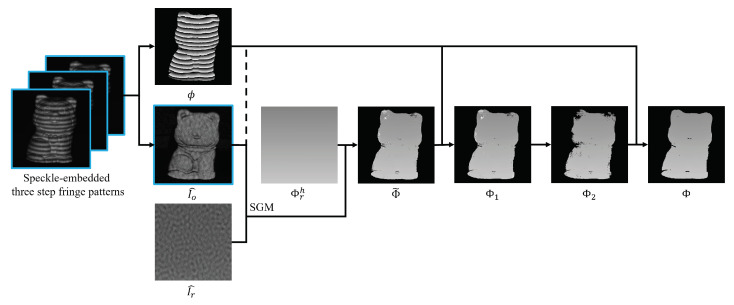
The overall computational framework of our proposed method.

The overall framework of our proposed method is summarized in Figure 4. In total, there were only three patterns projected. We obtained the wrapped phase ϕ and ${\hat{I}}_{o}$ from the speckle-embedded three-step phase-shifted fringe patterns. Then we utilized the correspondence between ${\hat{I}}_{o}$ and ${\hat{I}}_{r}$ found by SGM to extract the low-accuracy absolute phase $\tilde{\Phi }$, and directly unwrapped ϕ to obtain $\Phi_{1}$. Next, pixels in the discontinuous minor regions were masked out to generate $\Phi_{2}$. Finally, local spatial phase unwrapping unwraps the phase at pixels we masked out in the previous step with respect to $\Phi_{2}$. The resulting high-accuracy absolute phase Φ can be used for high-accuracy 3D reconstruction [30].

## 3. Results and Discussions

**Figure 5:**
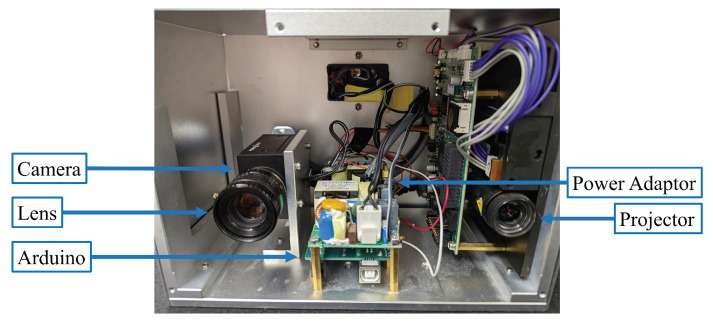
Photograph of our prototype system.

We verified our proposed method by developing a structured light prototype system, shown in Figure 5. The system was comprised of one camera (FLIR Grasshopper3 GS3-U3-23S6M) that was attached to a 16 mm focal length lens (Computar M1614-MP2) and one projector (Texas Instrument LightCrafter 4500). The full resolution of the camera was 1920×1200 pixels and the full resolution of the projector was 912×1140 pixels. The fringe period of the three-step phase-shifted fringe patterns was 18 pixels. The fringe periods of the multi-wavelength phase-shifting algorithm were 36, 216, and 1140 pixels for the horizontal direction and 18, 114, and 912 pixels for the vertical direction. The system was calibrated using Zhang and Huang’s method [30] and the camera coordinate system was chosen to be the world coordinate system. The subset size for calculating $C_{ZNSSD}$ in the SGM algorithm was 31×31 pixels, and the $\hat{\gamma }$ and $\hat{I^{\prime \prime }}$ thresholds were 0.1 and 3, respectively. The minimum area for a connected region was the same as the number of pixels of a $C_{ZNSSD}$ subset, 31×31 pixels.

**Figure 6:**
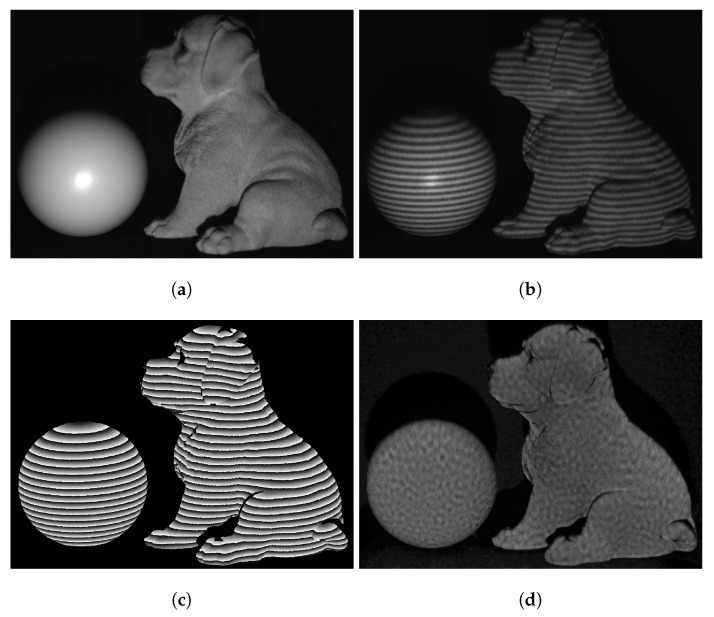
Measurement of two isolated 3D objects. (**a**) Photograph of the objects. (**b**) One of the fringe images. (**c**) Wrapped phase ϕ. (**d**) Recovered speckle image ${\hat{I}}_{o}$.

We compared the computational framework in this work with our previously developed DIC-assisted method [25] and the multi-wavelength phase unwrapping method. The grid step in the DIC-assisted method was set to 1 pixel in order to perform pixel-wise searches. We measured two isolated 3D objects: a sphere and a complex sculpture, shown in Figure 6a. Prior to any 3D measurement, we obtained ${\hat{I}}_{r}$ and its corresponding $\Phi_{r}^{h}$ and $\Phi_{r}^{v}$. Then, we measured 3D objects by projecting speckle-embedded three-step phase-shifted fringe patterns and capturing these fringe patterns. Figure 6b shows one of the fringe images. The wrapped phase ϕ and the speckle image ${\hat{I}}_{o}$ retrieved from the fringe images are shown in Figure 6c and Figure 6d, respectively.

**Figure 7:**
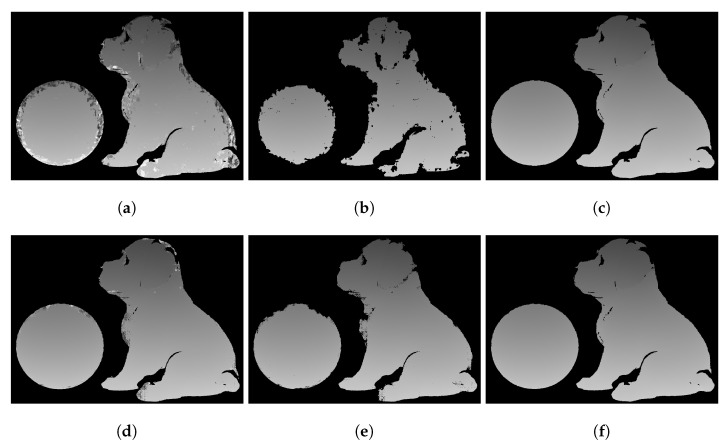
Phase unwrapping process of the 3D objects. (**a**) Low-accuracy absolute phase generated from the DIC-assisted method. (**b**) Partially unwrapped phase map using (**a**) after removing error points. (**c**) Final high-accuracy absolute phase map from the DIC-assisted method. (**d**) Low-accuracy absolute phase map, $\tilde{\Phi }$, generated from the proposed method. (**e**) Partially unwrapped phase map, $\Phi_{2}$, using (**b**) after removing error points. (**f**) Final high-accuracy absolute phase map, Φ, using the proposed method.

**Figure 8:**
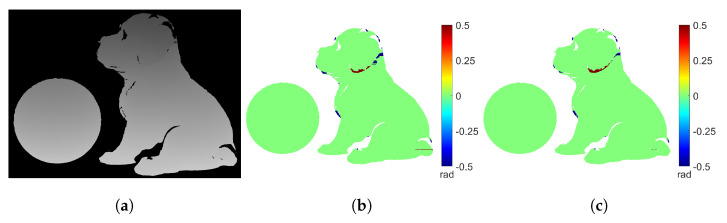
Comparison of the absolute phase maps obtained from unwrapping ϕ with the DIC−assisted method, our proposed method, and the multi−wavelength method. (**a**) Absolute phase map from the multi−wavelength method. (**b**) Difference map between Figure 7c and (**a**) (3615 error points). (**c**) Difference map between Figure 7f and (**a**) (2997 error points).

The wrapped phase ϕ was then unwrapped by the three aforementioned different methods. The results are shown in Figure 7 and Figure 8. Figure 7a shows the low-accuracy absolute phase map generated from the DIC-assisted method. Figure 7b shows the partially unwrapped phase map after error removal using Figure 7a. Figure 7c shows the final high-accuracy absolute phase map using the DIC-assisted method. Figure 7d shows the low-accuracy absolute phase map $\tilde{\Phi }$ generated from the proposed SGM. Figure 7e shows the partially unwrapped phase map ${\tilde{\Phi }}_{2}$ using Figure 7d. Figure 7f shows the final high-accuracy absolute phase map Φ using our proposed method. From Figure 7a,d, one can see that the SGM algorithm results in a much better quality correspondence map, especially near the edges. Therefore, it is not required to mask out the edges, so more pixel correspondences are preserved in Figure 7e compared to Figure 7b. As a result, the local spatial phase unwrapping algorithm is required on much fewer points. Furthermore, the DIC finds correspondences at an average rate of 10 pixels/s but the SGM finds correspondences at an average rate of 320 pixels/s, which is over 30 times faster.

For comparison, the absolute phase map obtained from the multi-wavelength method is shown in Figure 8a. Figure 8b shows the difference map between Figure 7c and Figure 8a. Figure 8c shows the difference map between Figure 7f and Figure 8a. There are 3615 error points in Figure 8b and 2997 error points in Figure 8c. The proposed method generates similar or slightly fewer error points than the DIC-assisted method. Also, Figure 8c shows that our proposed method and the multi-wavelength method produce identical results in smooth areas. However, there are phase unwrapping errors near the abrupt surfaces (e.g., around the ear of the dog sculpture). This is because in these areas the speckle pattern comes from different heights. Therefore, it is difficult for the SGM algorithm to accurately determine the correct correspondence. Nevertheless, it is important to note that only a small number of pixels are unwrapped incorrectly in such a complex scene, which demonstrates the success of our proposed method.

**Figure 9:**
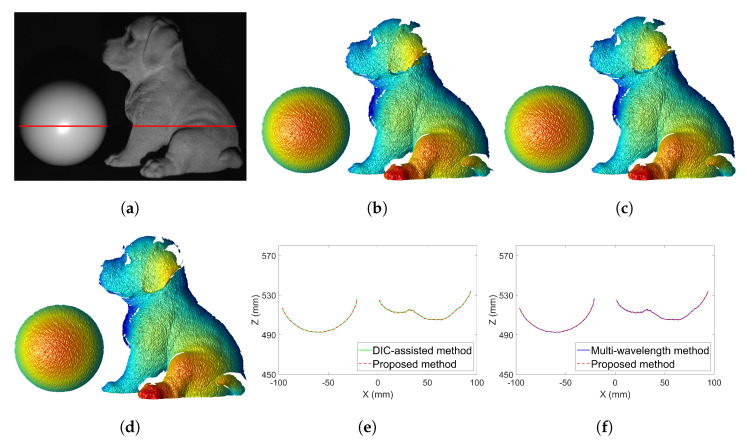
Three dimensional (3D) reconstructed shapes with the absolute unwrapped phase using different phase unwrapping methods. (**a**) Photograph of the 3D objects with the red line being the cross−section we examined. (**b**) 3D reconstruction using the unwrapped phase map shown in Figure 7c. (**c**) 3D reconstruction using the unwrapped phase map shown in Figure 7f. (**d**) 3D reconstruction using the unwrapped phase map shown in Figure 8a. (**e**) Overlap of the same cross−section of the reconstructed 3D shapes in (**b**,**c**). (**f**) Overlap of the same cross−section of the reconstructed 3D shapes in (**c**,**d**).

Figure 9 shows the 3D reconstructions from the unwrapped phase with the DIC-assisted method, the proposed method, and the multi-wavelength method. We can observe that our proposed method successfully reconstructed the details of the dog sculpture including edges as the other two methods. Figure 9e,f shows the overlapping cross-section of reconstructed 3D shapes. As expected, the results from the proposed method and the other two methods are identical in the smooth areas. One may notice that 3D reconstructions have large random noise due to the embedded speckle pattern.

**Figure 10:**
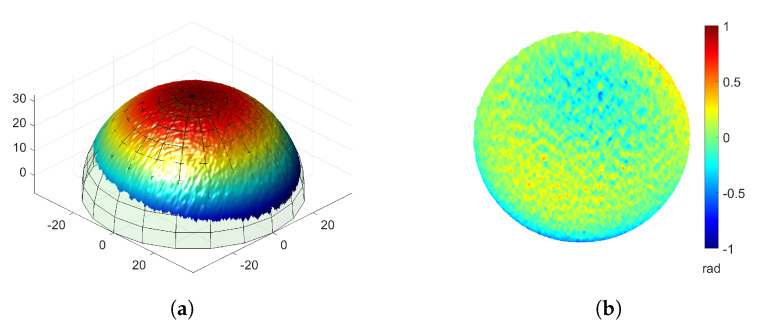
Accuracy evaluation of the measuring result of a sphere. (**a**) Overlap 3D measured data of the sphere shown in Figure 9c with an ideal sphere. (**b**) Error map of (**a**) (RMS: 0.13 mm, Mean: $-0.23\;\times \;10^{-3}\;mm$, σ: 0.13 mm).

We further evaluated the measurement accuracy of our proposed method. An ideal sphere was fitted to the point cloud of the measured sphere shown in Figure 9c. A Gaussian filter with a size of 5×5 pixels and a standard deviation of 5/3 pixels was applied to the measured data to reduce the most significant random noise. The function of the ideal sphere is

(12) $${\left(x-x_{0}\right)}^{2}+{\left(y-y_{0}\right)}^{2}+{\left(z-z_{0}\right)}^{2}=r^{2},$$

where $\left(x_{0},y_{0},z_{0}\right)$ is the center of the sphere and *r* is the radius of the sphere. The error map was created by taking the difference between the measured data and the ideal sphere. Figure 10a shows the overlap of the measured data and the ideal sphere. Figure 10b shows the error map. The root mean square (RMS) error is approximately 0.13 mm and the mean of the error is $-0.23\;\times \;10^{-3}\;mm$ with a standard deviation (σ) of 0.13 mm. These are quite small compared to the radius of the sphere (approximately 39.51 mm).

**Figure 11:**
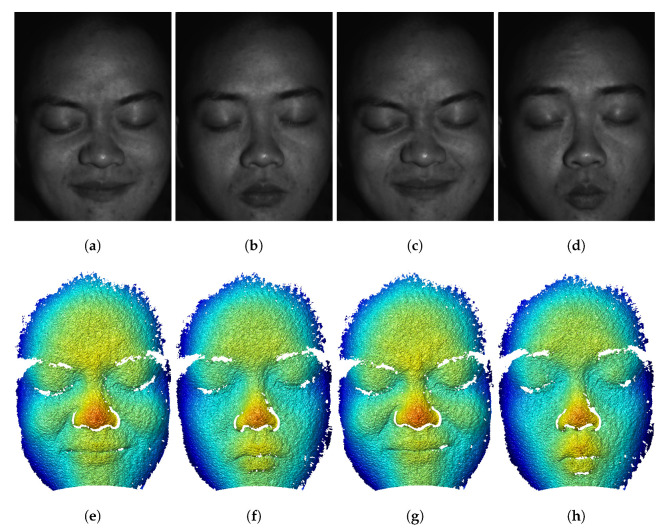
Typical 3D frames of capturing a moving face at 100 Hz (Video S1). (**a**–**d**) Texture images of the face. (**e**–**h**) 3D geometry of (**a**–**d**).

Since only three patterns are required for each 3D reconstruction, we also demonstrated the high-speed ability of the proposed method by capturing a dynamic human face. We used the same structured light system and changed the camera resolution to 800×600 pixels in order to increase the camera capture rate to 300 Hz. The projector’s refresh rate was also set to 300 Hz. The $\hat{\gamma }$ threshold is increased to 0.18. Since we only need three patterns to reconstruct one 3D frame, the achieved 3D measurement speed is 100 Hz. Figure 11 shows a few typical frames of 3D reconstructions shown in Video S1. This experiment demonstrated the success of our proposed method for measuring dynamic scenes with complex surface geometry and texture.

## 4. Conclusions

In this research, we proposed an SGM-assisted absolute phase unwrapping method on a single-camera and single-projector structured light system. The proposed method can measure the absolute depth of multiple isolated 3D objects with complex geometries without prior knowledge of the scene. Compared to our previously developed DIC-assisted method, the SGM-assisted method is more than 30 times faster in pixel-wise correspondence search and generates many fewer correspondence errors. This enabled us to successfully reconstruct one 3D frame using only three speckle-embedded phase-shifted patterns. The proposed method achieves a high measurement accuracy: an RMS error of 0.13 mm with a mean of $-0.23\;\times \;10^{-3}\;mm$ and a standard deviation of 0.13 mm for a sphere with a radius of approximately 39.51 mm using the speckle-embedded fringe pattern. Since only three patterns are required for one 3D reconstruction, the proposed method can achieve a high speed. We developed a high-speed prototype system that achieved a 100 Hz 3D measurement rate.

## Data Availability

Data underlying the results presented in this paper are not publicly available at this time but may be obtained from the authors upon reasonable request.

## Supplementary Materials

The following supporting information can be downloaded at: https://www.mdpi.com/article/10.3390/s23010411/s1, Video S1: Captured 3D video of a moving face at 100 Hz.
